# Characterization using ultra-deep sequencing of the intra-host distribution of the mutations associated with *H. pylori* antibiotic resistance

**DOI:** 10.1186/s12941-025-00840-5

**Published:** 2026-01-22

**Authors:** Laura Chaufour, Alexandra Herve, Birama Ndiaye, Lucie Karayan-Tapon, Médéric Briand, Frédérique Lartigue, Christophe Burucoa, Maxime Pichon

**Affiliations:** 1 CHU de Poitiers, Département des Agents Infectieux, Laboratoire de Bactériologie, Poitiers, France; 2 INSERM U1070 PHAR2 Pharmacology of Antimicrobial Agents and Resistances, Université de Poitiers, Poitiers, France; 3 CHU de Tours, Laboratoire de Bactériologie-Virologie-Hygiène, Tours, France; 4 CHU de Poitiers, Laboratoire de Cancérologie Biologique, Poitiers, France; 5 Université de Poitiers , UR24144, PRODICET, Poitiers, France; 6 Université de Tours, INRAe, UMR1282 Infectiologie et Santé Publique, Tours, France

**Keywords:** *Helicobacter pylori*, Gastric biopsies, Antibiotic resistance, Bacterial subpopulation, Sequencing

## Abstract

**Introduction:**

*Helicobacter pylori* is a slow-growing, gram-negative strictly pathogenic bacterium, which colonizes the stomachs of half the global population and is responsible for gastritis, peptic ulcer and even adenocarcinoma, Treatment of choice for eradication is a combination of PPIs and multiple antibiotic therapy. Recently, therapeutic failures began to be attributable to increased antibiotic resistance due to mutations in identified genes *(rpoB*, 16S rRNA coding gene, *gyrA*, 23S rRNA coding gene, *pbp1A*,* frxA*,* rdxA)*.

**Objectives:**

This study aimed to determine, using ultra-deep sequencing, the distribution of mutations in patient s hospitalized or undergoing screening for *H. pylori*.

**Methods:**

Gastric biopsies were obtained from two different anatomical regions (antrum/fundus) in 18 patients’ samples from 1998 to 2021, in four French hospitals. Following automated extraction, DNA of *H. pylori* was amplified using multiplexed PCR, before sequencing on the Illumina iSeq100 platform.

**Results:**

Antral diversification of *H. pylori* populations is significantly greater than that at the fundic level for *rpoB* and *rdxA*. Fundic diversification of *H. pylori* populations is significantly greater than that at the antral level for the 23S rRNA coding, *rdxA* and *rpoB* genes (*p* < 0.05), with inter-individual variation.Conversely, the 16S rRNA, frxA, *gyrA* and pbp1A genes exhibited no significant variation (*p* > 0.05).

**Discussion:**

This first study using in-house high-throughput sequencing of *H. pylori* on clinical biopsies from the same patients reinforces the hypothesis that the bacterial population within the same host is heterogeneous. The presence of minority variants justifies the need for at least two biopsies to ensure robust testing of the *H. pylori* antibiotic susceptibility profile.

**Supplementary Information:**

The online version contains supplementary material available at 10.1186/s12941-025-00840-5.

## Introduction

*Helicobacter pylori*, a microaerophilic, gram-negative, slow-growing, spiral-shaped, and flagellated bacterium is a major public health problem, affecting approximately half of the world’s population [1]. The prevalence of infection is significantly higher in developing countries (85%) than in Europe or North America (30–40%) [2]. In France, *H. pylori* prevalence is estimated at 30%, and increases with age [3].

*H. pylori* is the first bacterium to have been formally recognised as a carcinogen, with the capacity to cause a variety of gastrointestinal illnesses, including gastric adenocarcinoma, and gastric mucosa-associated lymphoid tissue lymphoma [4]. The lifetime risk of developing adenocarcinoma in an individual infected with *H. pylori* is estimated at 1–4% [4–6]. Gastric cancer is the fourth most diagnosed cancer, and it has a particularly poor prognosis, with a 5-year relative survival rate of only 31%. In 2023, it was the second leading cause of cancer-related mortality worldwide [7]. Treatment of the infection is effective in healing the ulcer and preventing the development of gastric adenocarcinoma.

In terms of treatment, in 2017 the French Health Authority (HAS) recommended a 14-day quadruple therapy comprising Proton-Pump Inhibitor (PPI), amoxicillin, clarithromycin, and metronidazole as a first-line treatment option. Alternatively, a 10-day quadruple therapy comprising PPI, bismuth salt, tetracycline, and metronidazole was recommended. In cases where the antibiogram is taken into consideration, triple therapy with clarithromycin, amoxicillin, and PPI is employed. However, in instances involving resistance to clarithromycin, triple therapy with levofloxacin, amoxicillin, and PPI is employed [3].

*H. pylori* acquires resistance to antibiotics primarily through point mutations, predominantly in genes encoding antibiotic targets. The mutation rate has been estimated at 10⁴/nucleotide/year, higher than that of most bacteria (“hypermutating" bacterium) [8]. Furthermore, *H. pylori* is a naturally competent bacterium, capable of internalizing DNA from the external environment and integrating it into its genome through homologous recombination [9]. *H. pylori* infections have been shown to be multifaceted, with contamination occurring through either multiple strains of varying origins and resistances ("multiple" infection) or a single strain diversifying to produce various isolates ("mixed" infection).

Clarithromycin, a macrolide antibiotic, belongs to a family of bacteriostatic antibiotics, by binding irreversibly to a component of the bacterial ribosome. Mutations in the loop of 23S rRNA can lead to reduced effectiveness of macrolide antibiotics and the emergence of resistance. Most resistant strains exhibit mutations A2142G/C and A2143G (A2146G/C and A2147G depending on the nomenclature, respectively) [10, 11]. Levofloxacin, a fluoroquinolone antibiotic, is a bactericidal drug that inhibits bacterial topoisomerase. In *H. pylori*, numerous mutations in the *gyrA* gene have been identified as the underlying causes of fluoroquinolone resistance [12–14]. As a member of the imidazole group, metronidazole is a bactericidal antibiotic, of which the precise mechanism of action remains unclear, implicating the formation of components inhibiting nucleic acid synthesis. The presence of mutations in the *rdxA* gene (which encodes a nitroreductase) has been described as a criterion insufficient to confer resistance to metronidazole leading, to exploration of the role of *frxA* in *H. pylori* proliferation [15]. Amoxicillin, a bactericidal antibiotic belonging to the penicillin group, impedes bacterial cell wall synthesis, leading to bacterial lysis. Resistance to amoxicillin is attributed to mutations in the *plp1A* gene, even though this phenomenon is an exceedingly uncommon occurrence [16]. Rifamycin, a member of the rifampicin group, is a bactericidal antibiotic that binds to the beta subunit of DNA-dependent RNA polymerase, thereby inhibiting transcription. The beta subunit is encoded by *rpoB* [17]. Lastly, classified as a cyclin group, tetracycline is a bacteriostatic antibiotic impeding protein synthesis, by affecting 16S rRNA [18].

Since its initial identification, *H. pylori* has demonstrated increasing resistance to antibiotics, with multidrug resistance reaching 8%. Strains resistant to rifamycin, tetracycline, and amoxicillin have recently emerged [19]. In France, data concerning resistance to the various antibiotics employed in eradication therapy have been published by Megraud et al. based on European data from 2018 [20]. As demonstrated by french data (which constitute more than 15% of the data in the article), resistance to clarithromycin is observed in 22.5% of the strains analysed, which equates to almost three defined daily doses per 1000 inhabitants per day. Furthermore, resistance to levofloxacin has been documented in 19.2% of the analysed strains, which is commensurate with approximately two defined daily doses per 1000 inhabitants per day. More recently, it is important to note that, based on data reported for 1330 strains from 27 laboratories in the first half of 2025, primary resistance to clarithromycin, levofloxacin, rifampicin, and tetracycline is 26%, 19.7%, 1.2%, and 0% respectively (HELICONet network data, unpublished data). This resistance is responsible for the rise in therapeutic failures of triple therapy (PPI-amoxicillin-clarithromycin), thereby justifying priority-guided treatment.

In this context, the objective of the present study was to characterise the intra-host distribution of antibiotic resistance in *H. pylori* strains using Next Generation Sequencing (NGS), in view of understanding the development of the dramatic epidemiology of antibiotic resistance. This information could support or refute the need for multiple biopsies, as demonstrated by previous research focusing on clarithromycin-resistant strains using PCR only.

## Methods

### Study population

**Fig. 1:**
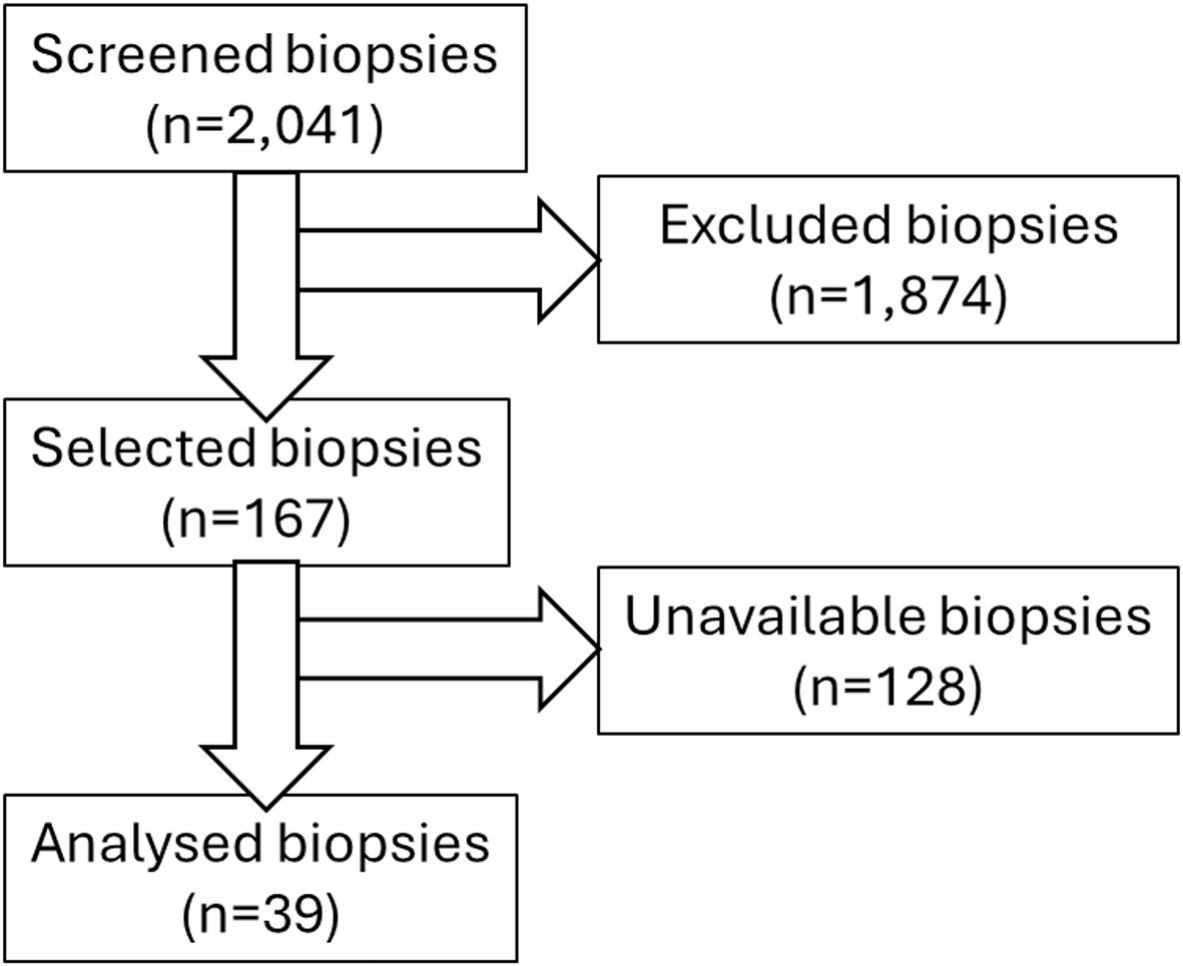
Flow chart for selection

A retrospective, multicentric study was conducted including patients who were hospitalised or consulted for endoscopy at University Hospital of Poitiers, and the hospitals of Niort, Laval, and Angouleme, between 1998 and 2021. During the specified period, 2041 biopsies were received in the laboratory, of which 2002 were excluded from the study due to inclusion criteria requiring a minimum of two separated biopsies per patient (Fig. 1). The final cohort comprised 39 intra-host biopsies, (i.e. sampled from different parts of the same subject during the same endoscopy procedure).

Following the sampling process, the gastric biopsies were subjected to mechanical grinding (MM400, Retsch, Haan, Germany) and subsequently divided into three aliquots for i/multiplex real-time PCR amplification (Amplidiag *H. pylori* + clariR, Mobidiag, Espoo, Finland) targeting the 23s rRNA gene so as to determine the presence or absence of resistance-associated mutations (A2142G/C and A2143G); ii/ cultivation of the sample in a microaerophilic atmosphere at 35 ± 2 °C for a period of 48 to 72 h for antibiotic susceptibility testing in accordance with national recommendations; and iii/immediate freezing at − 80 °C. Note that this aliquot was not subjected to any thawing processes prior to the analysis described thereafter.

### Experimental control

A positive control (consisting of a J99 strain (ATCC 70082)) and a negative control (consisting of lysis buffer and proteinase K) were extracted and analysed in a manner consistent with the other samples. The reference strain J99 possesses no antibiotic resistance, and its genome has been fully sequenced. Consequently, it was employed as a negative control for bioinformatic analysis. The mutations identified in this strain reveal the variability of the *H. pylori* genome compared with another reference strain, ATCC 26695, which has the same characteristics as strain J99 (absence of antibiotic resistance and fully sequenced genome), allowing for standardized comparison with the literature The negative control was used to validate the extraction, amplification and sequencing processes, thereby excluding the possibility of contamination by other samples. The bioinformatics component was likewise validated by this control, thereby reinforcing the observations made during the sequencing analysis (possible mutations found at the time of data analysis were excluded).

### Nucleic acid isolation

Nucleic acid isolation process was carried out as follows: following a gentle thawing procedure by placing it at + 4 °C, all biopsies were extracted with the EasyMag system (bioMérieux, Marcy l’étoile, France) following digestion for 30 min at 65 °C. Concentration of DNA in the extracts was then quantified with the Qubit dsDNA HS Assay Kit (Thermofisher scientific, Waltham, MA, USA) using a Qubit 4.0 fluorometer (Thermofisher scientific) to quantitatively validate the extraction process.

### Pre-library amplification

The amplification of PCR reactions was achieved through utilisation of a multiplex PCR method that had been developed in-house (Suppl. Table 1). The amplification of seven targets was accomplished by employing primers sourced from the KAPA Hifi HotStart PCR kit (Roche, Basel, Switzerland) in a CFX96 system (BioRad, Hercules, CA, USA). Each reaction well contained 2.5 µl of DNA, 10 µl of primers at a concentration of 1 mM, and 12.4 µl of mix PCR. The thermal protocol comprised one cycle consisting of 95 °C for 3 min, followed by 35 cycles comprising three steps: (i) 98 °C for 20 s, (ii) 69 °C for 15 s, and (iii) 72 °C for 2 min. Amplicons were purified using magnetic beads (Machery Nagel, Hoerdt, France) and verified by electrophoresis in agarose gel (1.5%) after migration for 45 min.

### Library preparation and sequencing

The DNA was tagged with the Nextera XT Index Kit (Illumina, San Diego, California, USA) and the Nextera DNA Sample Preparation Kit (Illumina) using the Nextera XT Index Kit 24 samples (Illumina). Subsequently, the library was purified using magnetic beads (Machery Nagel) and quantified using a Qubit 4.0 Fluorometer (Thermofisher Scientific). Subsequently, the library was multiplexed and diluted to 58 pM prior to loading onto the ISeq100 flow cell V1 in a 150 × 2 paired-end configuration (Illumina).

### Informatic and statistical analyses

The analysis was conducted using software developed locally, at the CHU, designated “SIDERANT”. In the initial step, the generated reads were analysed with the FASTQC program v0.12.1 to evaluate quality control checks on Reads (per base sequence quality, per sequence quality scores) and Cutadapt v1.18 to filter all Reads with mean Phred quality lower than 30. The filtered reads were aligned on the *H. pylori* genome ATCC26695 using BWA v0.7.17. The generated BAMs were subsequently indexed to prepare for the variant calling step, which was carried out using the custom-made program GRVC v1.7, which generates VCF files. Finally, the variants were annotated using SNPEff software v4.3. All annotated variants were then inserted into a MySQL database, which was connected to an Apache2 web interface to facilitate data exploration. As the primary objective of the present project was to describe the heterogeneity of the *H. pylori* subpopulations in a single patient, only mutations that met all the following criteria were considered: sequencing depth > 100X, absence of strand bias, presence/absence in one of the biopsies, non-synonymous mutation and absence in the ATCC reference strains. The sequences were submitted to the Sequence Read Archive database and are now accessible via the accession number: PRJNA1221844 (SRR32293500 to SRR32293595). The variables were analysed using Fisher Exact and Student Tests with GraphPad version 9.0 software. Statistical significances were determined by calculating the p-value, with a value less than 0.05 indicating statistical significance.

## Results

### Global results

**Fig. 2:**
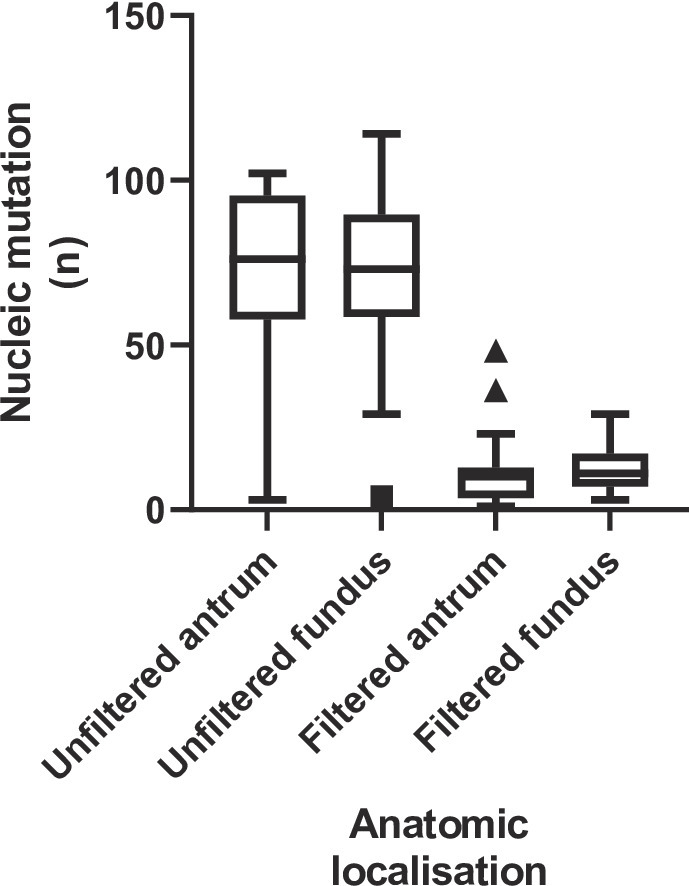
Anatomical comparison of genetic diversities

In this study, eighteen patients underwent transverse sampling, with an average of 2.17 samples per patient (Table 1). All patients underwent two biopsies, except for patients #1, #3 and #17, who underwent a third biopsy (without precision of anatomic localisation). During the data analysis, mutations were identified in the positive control strain. Regarding these mutations, the mutation rate induced by the techniques employed in the project was estimated at ten mutations per million nucleotides. Given that the mutations present in these strains do not contribute to antibiotic resistance, they were excluded from the mutation analysis. Given the potential influence of prior eradication treatment on the genetic diversification of *H. pylori*, a statistical comparison of genetic and anatomical diversities was performed according to this parameter. In the present cohort, no statistically significant differences could be identified, either globally or after stratification by analysed genes (*p* > 0.05). Global difference between antrum and fundus have been considered as non-significant (*p* > 0.05) (Fig. 2).

**Table 1:** Phenotypic characteristics of the analyzed samples

Patient	Anatomical location	Antibiotic susceptibility testing (MIC, mg/l)
#1	Site A	–
	Site B	Lev (0.125), Rif (0.125), Tet (0.032), Amx (< 0.016), *Cla (> 256)*,* Met (96)*
	Site C	–
#2^+^	Antrum	Lev (0.125), Rif (4), Tet (0.023), Cla (0.016), Amx (< 0.016), *Met (256)*
	Fundus	Lev (0.125), Rif (0.38), Tet (0.023), Amx (0.016), *Cla (96)*,* Met (> 256)*
#3	Antrum	Lev (0.064), Rif (0.75), Tet (0.064), Cla (< 0.016), Amx (< 0.016), *Met (> 256)*
	Fundus	Lev (0.125), Rif (1), Tet (0.125), Cla (0.032), Amx (< 0.016), *Met (> 256)*
	Angulus	Lev (0.125), Rif (1), Tet (0.125), Cla (0.023), Amx (< 0.016), *Met (> 256)*
#4	Antrum	Rif (0.5), Tet (0.023), Amx (0.016), *Lev (8)*,* Cla (12)*,* Met (> 256)*
	Fundus	Rif (0.5), Tet (0.023), Amx (0.023), *Lev (8)*,* Cla (32)*,* Met (> 256)*
#5^+^	Antrum	Rif (0.25), Tet (0.19), Cla (0.023), Amx (0.047), *Lev (6)*,* Met (> 256)*
	Fundus	Rif (1), Tet (0.25), Cla (0.064), Amx (0.094), *Lev (6)*,* Met (> 256)*
#6^+^	Antrum	Lev (0.094), Rif (0.38), Tet (0.032), Cla (< 0.016), Amx (< 0.016), *Met (> 256)*
	Fundus	–
#7	Antrum	Lev (0.094), Rif (0.38), Tet (0.094), Cla (0.016), Met (0.19), Amx (< 0.016)
	Fundus	Lev (0.19), Rif (0.38), Tet (0.032), Cla (0.032), Met (0.19), Amx (< 0.016)
#8^+^	Antrum	Lev (0.125), Rif (1), Tet (0.023), Cla (0.064), Amx (0.032), *Met (128)*
	Fundus	Lev (0.125), Rif (4), Tet (0.023), Cla (0.064), Amx (0.032), *Met (192)*
#9	Antrum	Lev (0.064), *Cla (> 256)*
	Fundus	Lev (0.064), Cla (< 0.016)
#10^+^	Antrum	–
	Fundus	Lev (0.094), Rif (0.19), Tet (0.016), Cla (0.032), Met (0.75), Amx (0.016)
#11	Antrum	Lev (0.125), Rif (0.047), Tet (0.023), Amx (< 0.016), *Cla (> 256)*,* Met (32)*
	Fundus	Lev (0.19), Rif (0.094), Tet (0.032), Amx (0.016), *Cla (> 256)*,* Met (128)*
#12	Antrum	Lev (0.125), Rif (0.125), Tet (0.064), Cla (0.064), *Met (96)*
	Fundus	Lev (0.094), Rif (0.19), Tet (0.064), Cla (0.064), Amx (0.016), *Met (48)*
#13	Antrum	Lev (0.094), Rif (1), Tet (0.023), Cla (0.023), Amx (0.016), *Met (16)*
	Fundus	Lev (0.125), Rif (1), Tet (0.032), Cla (0.047), Amx (0.016), *Met (16)*
#14^+^	Antrum	Lev (0.19), Rif (0.38), Tet (0.047), Cla (0.016), Amx (< 0.016), *Met (32)*
	Fundus	Lev (0.064), Rif (0.50), Tet (< 0.016), Cla (< 0.016), Amx (< 0.016), *Met (32)*
#15	Antrum	Lev (0.094), Rif (1), Tet (0.064), Cla (0.047), Amx (< 0.016), *Met (> 256)*
	Fundus	Lev (0.125), Tet (0.032), Cla (0.032), Amx (< 0.016), *Met (> 256)*
#16	Antrum	Lev (0.125), Rif (0.5), Tet (0.032), Amx (< 0.016), Met (1,5), *Cla (8)*
	Fundus	Lev (0.125), Rif (0.5), Tet (0.032), Amx (< 0.016), Met (1,5), *Cla (8)*
#17	Site A	Lev (0.125), Rif (2), Tet (0.032), Amx (< 0.016), *Cla (8)*,* Met (> 256)*
	Site B	Lev (0.125), Rif (1), Tet (0.023), Amx (< 0.016), *Cla (1)*,* Met (> 256)*
	Site C	-
#18^+^	Antrum	Lev (0.125), Rif (2), Tet (0.032), Cla (0.047), Amx (0.016), *Met (> 256)*
	Fundus	Lev (0.094), Rif (3), Tet (0.023), Cla (0.023), Amx (0.016), *Met (> 256)*

*MIC* Minimal inhibitory concentration, *Cla* Clarithromycin, *Lev* Levofloxacin, *Rif* Rifampicin, *Tet* Tetracycline, *Amx* Amoxicillin, *Met* Metronidazole, *Pyl* Pylera i.e. Metronidazole, Tetracycline and Bismuth salts. The symbol ‘+’ is used to denote patients who have been sampled after at least one eradication treatment. The antibiotics for which the tested strains showed resistance in MIC determinations have been italicized

### 23S rRNA

**Fig. 3:**
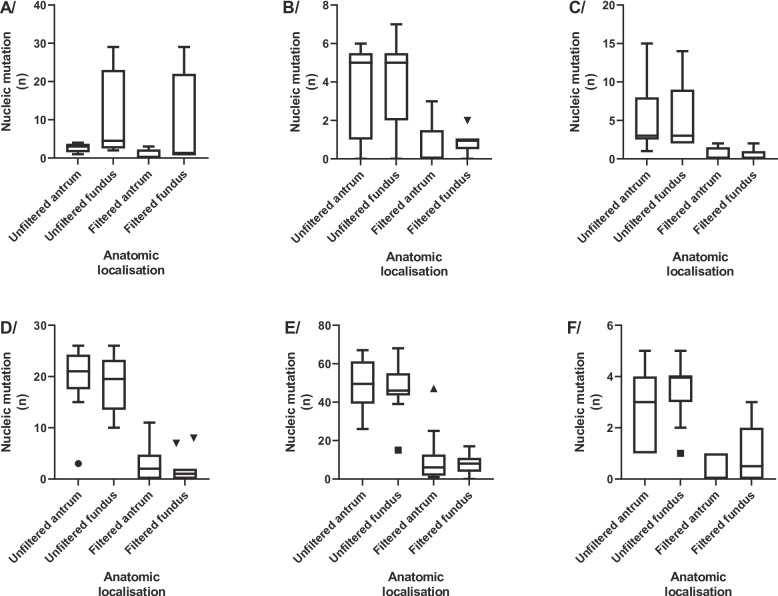
Anatomical comparison of genetic diversities depending on the considered gene before or after filtering of non-differentially represented mutation. **A/** 23S rDNA coding gene; **B/** *gyrA* gene; **C/** *frxA* gene; **D/** *rdxA* gene; **E/** *rpoB* gene ; and **F/** 16S rDNA coding gene

The 23S rRNA gene was successfully sequenced in seven patients (Patients #1, #3, #6, #7, #10, #12, #15) 7/18; 39%) (Fig. 3A and Suppl. Table 2) but for patients #10 and #12, only one of the two biopsies was sequenced, so they were excluded. In the patient #3, #6, #7 and #15 (Suppl. Table 3) the number of mutations identified in the fundus was significantly higher than that observed in the antrum (*p* < 0.05). However, when the frequency of each mutation was considered, variability between the fundus and the antrum could no longer be considered as significant (*p* > 0.05).

### *gyrA*

For the *gyrA* gene, a discrepancy was observed in nine out of the 14 successfully sequenced patients (9/14; 64,3%) (Patients #1, #4, #9, #10, #11, #12, #13, #14, #17) (Fig. 3B and Suppl. Tables 4 and 5). The number of mutations identified in the fundus was not significantly different from that observed in the antrum (*p* > 0.05). It is noteworthy that all mutations were present at a very low percentage (0,53% to 5.61%), indicating that they constituted a minority of the included patients.

### *frxA and rdxA*

The *frxA* gene was successfully sequenced in eleven patients (11/18; 61.1%). In seven of them (7/11; 63.6%) (Patients #1, #2, #4, #7, #10, #12, #14) (Fig. 3C and Suppl. Tables 6 and 7), subpopulations were found to differ between the antrum and fundus (with some mutations being observed only in one anatomical site). In general, however, there was no significant difference in mutation rates between the two sites (*p* > 0.05). For the *rdxA* gene, sixteen patients were correctly sequenced (16/18; 89%) (Patients #1, #2, #3, #4, #5, #7, #8, #9, #11, #12, #13, #14, #15, #16, #17, #18) (Fig. 3D and Suppl. Tables 8 and 9). One patient presented strictly the same mutational profile between anatomical sites (patient #14). On a global scale, the number of mutations identified in the antrum was significantly higher than in the fundus (*p* < 0.05), yet there was no significant difference between the antrum and the fundus when the frequency of each mutation was considered (*p* > 0.05). The two genes had a low number of mutations, suggesting a high cost of adaptation per mutation.

### *pbp1A*

As only two patients could be successfully analysed (2/18; 11.1%) (Patients #1 and #7), the *pbp1A* gene was not the focus of sufficient exploration (Suppl. Tables 10 and 11). Of note, no interpretation could be obtained from these unique patients.

### *rpoB*

The *rpoB* gene was successfully sequenced in almost all patients (17/18; 94.4%) (except for Patient #6) (Fig. 3E and Suppl. Tables 12 and 13). The number of mutations identified in the antrum was significantly higher than in the fundus (*p* < 0.05), without any difference between the anatomical regions in the frequency of each mutation (*p* > 0.05). Furthermore, only five patients exhibited minority subpopulations (5/17, 29.4%), confirming that, as was observed for *frxA* and *rdxA* genes, mutations could not be observed without a high fitness cost in *rpoB* as well.

### 16S rRNA

The 16S rRNA coding gene demonstrated a 94.4% success rate of analysis in the 17 included patients (17/18) (Fig. 3F and Suppl. Tables 14 and 15). Among these patients, twelve (12/17; 70.6%) (Patients #1, #2, #3, #5, #9, #10, #11, #12, #13, #14, #15, #18) exhibited heterogeneity in nature (*p* < 0.05), while the number of fundic and antral mutations remained consistent (*p* > 0.05). Numerous minority variants were observed, without any significant differences between regions (*p* > 0.05).

## Discussion

*Helicobacter pylori* is a bacterium with a remarkable physiology. Pronouncedly ubiquitous, it constitutes a significant public health concern, contributing to the development of gastritis and ulcers and, most notably, playing a causal role in the development of gastric cancer [4]. Eradication of this bacterium has become increasingly complex with reinforced resistance to the usual antibiotics. The French National Authority for Health advocates a guided treatment approach based on antibiogram results, with a focus on clarithromycin and levofloxacin sensitivity [3]. Previous literature has documented mutations in antibiotic target genes as a cause of therapeutic failure. For instance, it has been clearly established that A2142G and A2143G (or A2146G and A2143G respectively) mutations in the 23S rRNA coding gene are associated with a high MIC to macrolides when clarithromycin is used [10, 11]. Similarly, N87T/I mutations in the *gyrA* gene are associated with a high MIC to quinolones when levofloxacin is administered [13, 14]. It is imperative to acknowledge the potential for disparities among antibiotic molecules of the same family, contingent on the specific mutation under scrutiny [13]. This observation underscores a need for further investigation on the role of the mutation in antibiotic resistance [21].

Furthermore, the mutational profile of biopsies from two anatomical sites (antrum/fundus) from each patient was found to differ. In a limited number of patients, *rpoB*, 23S rRNA coding gene and *rdxA* demonstrated antro-fundic diversity in terms of number of mutations between these sites. Conversely, the 16S rRNA coding gene, *frxA*, *gyrA* and *pbp1A* genes exhibited no significant variation. The observed discrepancies can be attributed to the distinct functions of these genes. Conversely, this alone does not provide a sufficient explanation for the differentiated distribution of these evolutionary characteristics, and the issue warrants future exploration via in vitro/in vivo/ex vivo analyses. The 16S rRNA coding gene, *gyrA* and *frxA* genes are considered as essential for bacterial physiology, suggesting that mutations in these genes are rapidly deleterious and incur a high fitness cost. The 16S rRNA coding gene code for a significant component of protein synthesis comprises ten regions universally present in all bacterial species [18]. The *gyrA* gene, in particular, is responsible for encoding an enzyme critical for bacterial chromosome replication (DNA supercoiling) [14]. Finally, the *frxA* gene is responsible for coding an enzyme essential for resisting phagocytosis in macrophages and reducing oxidative stress, both of which are vital for survival [15].

However, analysis of the frequencies of each mutation revealed no further variability between antrum and fundus, thereby supporting the hypothesis of the presence of very small minority populations in the fundus and antrum, as previously demonstrated by qPCR [22]. In the absence of mixed infection, and if a founder effect constitutes the observed populations, the initial population of *H. pylori* colonizing the gastric mucosa will diversify into different populations, each of them colonizing a distinct anatomic site. The potential value of testing this approach in stool samples is worthy of further consideration, insofar as they may constitute a concentrator of different subpopulations, thereby enhancing analytical sensitivity in the detection of antibiotic resistance mutations [23, 24].

This study did not demonstrate co-infection with two different strains (mixed infection) in any patient, a finding consistent with the clinical observation that puncture at several sites remains essential, as each biopsy provides different information.

However, in the present study the heterogeneity of resistance gene coverage and depth limits interpretation of the results. For instance, the *pbp1* gene and 23S rRNA coding gene were not covered for the entire cohort. Two possible approaches are available to overcome this limitation: the first entails use of a higher-throughput sequencing platform, from Illumina (NextSeq550, HiSeq, NovaSeq) or another manufacturer (Oxford Nanopore Technologies) [25]. Alternatively, the library production protocol could be optimised, either by increasing primer concentration or by enhancing the concentration balance of post-PCR amplicons. The second solution is currently preferred, due to a wish to maintain this approach on affordable sequencers (thousands of euros for the iSeq100 and for the MinION, as opposed to the NextSeq550, HiSeq, Novaseq, which costs several hundred thousand euros) for routine diagnostic use, due to its reduced bias (due to the potential impact of minority variants). The observed failure rate can be attributed to various factors, including low bacterial load (as no assessment of the amount of DNA per gene was conducted, with an overall quantification of the DNA present being performed after extraction and before library preparation/sequencing analysis), low DNA quality (as no assessment of DNA quality was conducted before sequencing analysis) or mutation in the primers used to amplify the nucleotide region to be sequenced, justifying the need for analytical checkpoints in further studies. The differential impact on different gene targets could be explained by two different reasons. The initial hypothesis, whilst less substantiated, remains a possibility. It is associated with the relative number of copies of each gene present in the bacteria under investigation (for example, a greater number of copies for 23S rRNA coding genecopies than pbp1A). The second hypothesis is more plausible and can be attributed to the design of the adopted primers. It is evident that, despite being designed in silico against *H. pylori*-specific genome sequences of various types and tested in vitro against bacterial reference strains (ATCC 26695 and J99 reference strains), it remains possible that the amplification efficiency of the primers and sensitivity/specificity may be inadequate. Exploration of these points in greater depth is imperative for future studies.

On the other hand, the relatively large cohort size (*n* = 18 phenotypically characterized patients resulting in 37 biopsies) is one of the strengths of this study, along with the quantity of data generated, as it enables anatomical heterogeneity to be highlighted. Nevertheless, given the significant length of the inclusion period (23 years), the number of case numbers could also be relatively low. This was due to the fact that patients had not been separately biopsied, as both the two or more biopsies were frequently pooled. The present data could highlight the benefit of separating these biopsies.

## Conclusion

In conclusion, the present study demonstrates a need to consider the intra-host diversity of *H. pylori* infection. The data presented herein contribute in several ways to the understanding of the large and complex problem of antibiotic resistance development. Firstly, it is important to note that the development of antibiotic resistance involves a stage of microbial population diversification, beginning with subpopulations that need to be characterised. Furthermore, it is important to understand the anatomically and genetically homogeneous and/or heterogeneous distribution of these resistant subpopulations to more finely tailor management, whether diagnostic (biopsy site) or therapeutic (choice of eradication antibiotic therapies). Finally, although this approach is becoming increasingly widespread, it is interesting to note that it highlights the interest and technical feasibility of studying minority subpopulations in the context of *H. pylori* infection, paving the way for further studies (longitudinal studies, therapeutic trials, etc.). A similar approach, utilising in-house or commercially available assays, would facilitate the analysis of both antibiotic resistance and virulence genes. These genes could then be contextualised with their genetic underpinnings, which are contingent on the phylogenetic nature of the sequenced strains. Finally, the present study could be pivotal in comprehending the development of antibiotic resistance in a specific geographical region and the disparities observed, extending above and beyond the simple socio-demographic distribution of antibiotic molecules.

## Supplementary Information

Below is the link to the electronic supplementary material.

Supplementary Material 1. Supplementary Table 1. Primers sequence. Supplementary Table 2. 23S rRNA gene diversification before filtering. * ND: not determined; R: Resistant; S: Susceptible; All mutations/modifications have been presented with number of reads and fraction of the respective subpopulation. Supplementary Table 3. 23S rRNA gene diversification after filtering. * ND: not determined; R: Resistant; S: Susceptible; All mutations/modifications have been presented with number of reads and fraction of the respective subpopulation. Supplementary Table 4. gyrA gene diversification before filtering. * ND: not determined; R: Resistant; S: Susceptible; All mutations/modifications have been presented with number of reads and fraction of the respective subpopulation. Supplementary Table 5. gyrA gene diversification after filtering. * ND: not determined; R: Resistant; S: Susceptible; All mutations/modifications have been presented with number of reads and fraction of the respective subpopulation. Supplementary Table 6. frxA gene diversification before filtering. * ND: not determined; R: Resistant; S: Susceptible; All mutations/modifications have been presented with number of reads and fraction of the respective subpopulation. Supplementary Table 7. frxA gene diversification after filtering. * ND: not determined; R: Resistant; S: Susceptible; All mutations/modifications have been presented with number of reads and fraction of the respective subpopulation. Supplementary Table 8. rdxA gene diversification before filtering. * ND: not determined; R: Resistant; S: Susceptible; All mutations/modificationss have been presented with number of reads and fraction of the respective subpopulation. Supplementary Table 9. rdxA gene diversification after filtering. * ND: not determined; R: Resistant; S: Susceptible; All mutations/modifications have been presented with number of reads and fraction of the respective subpopulation. Supplementary Table 10. Pbp1 gene diversification before filtering. * ND: not determined; R: Resistant; S: Susceptible; All mutations/modifications have been presented with number of reads and fraction of the respective subpopulation. Supplementary Table 11. Pbp1 gene diversification after filtering. * ND: not determined; R: Resistant; S: Susceptible; All mutations/modifications have been presented with number of reads and fraction of the respective subpopulation. Supplementary Table 12. rpoB gene diversification before filtering. * ND: not determined; R: Resistant; S: Susceptible; All mutations/modifications have been presented with number of reads and fraction of the respective subpopulation. Supplementary Table 13. rpoB gene diversification after filtering. * ND: not determined; R: Resistant; S: Susceptible; All mutations/modifications have been presented with number of reads and fraction of the respective subpopulation. Supplementary Table 14. 16S rRNA gene diversification before filtering. * ND: not determined; R: Resistant; S: Susceptible; All mutations/modification have been presented with number of reads and fraction of the respective subpopulation. Supplementary Table 15. 16S rRNA gene diversification after filtering. * ND: not determined; R: Resistant; S: Susceptible; All mutations/modifications have been presented with number of reads and fraction of the respective subpopulation.

## Data Availability

The sequences have been submitted to the Sequence Read Archive (SRA) database and are accessible via the accession number: PRJNA1221844 (SRR32293500 to SRR32293595).
